# 577. COVI-VAC™, a Live Attenuated COVID-19 Vaccine, Provides Single Dose Protection Against Heterologous Challenge with SARS-CoV-2 Beta (B.1.351) in the Syrian Golden Hamster Model

**DOI:** 10.1093/ofid/ofab466.775

**Published:** 2021-12-04

**Authors:** Anna Kushnir, Steffen Mueller, Sybil Tasker, J Robert Coleman

**Affiliations:** 1 Codagenix, Inc.; 2 Codagenix Inc., Farmingdale, New York

## Abstract

**Background:**

Although multiple COVID-19 vaccines are currently in use, emergence of novel SARS-CoV-2 variants with reduced neutralization raises concern of future vaccine escape. COVI-VAC™ is a live attenuated SARS-CoV-2 strain based on WA/1 being developed as an intranasal COVID-19 vaccine. COVI-VAC is attenuated through removal of the furin cleavage site and introduction of 283 silent, deoptimizing mutations that maintain viral amino acid sequence but slow viral replication in vivo by up to 5 logs. Notably, COVI-VAC presents all viral antigens in their native conformation and is not limited to spike. COVI-VAC demonstrated attenuation, immunogenicity and single dose protection in both the Syrian golden hamster and non-human primate models and currently in Phase 1 clinical trials. In this study, we evaluated efficacy of COVI-VAC against challenge with the Beta/B.1.351 variant in Syrian golden hamsters.

**Methods:**

Syrian golden hamsters, 7-10 weeks of age were, vaccinated intranasally with 8.25x10^4^ PFU COVI-VAC (n=28) or vehicle control (n=16). Twenty seven days post-vaccination, animals were challenged intranasally with 3x10^4^ PFU of wildtype (WT) SARS-CoV-2 Beta. Animals were weighed daily. Further analysis is being conducted with serum and key tissues from pre and post challenge timepoints to include neutralizing antibody, biodistribution (subgenomic qPCR) and histopathology.

**Results:**

COVI-VAC prevented weight loss following challenge with the heterologous variant of SARS-CoV-2, B.1.351/Beta (Figure). Results of additional analyses will be available before the IDWeek meeting.

Change in Weight following SARS-CoV-2 Beta Challenge

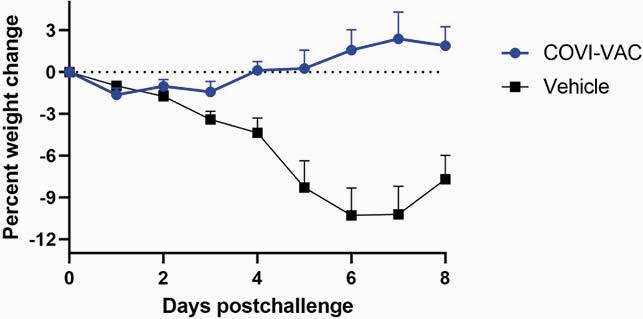

**Conclusion:**

COVI-VAC is protective against heterologous challenge with SARS-CoV-2 Beta. By presenting all viral antigens, COVI-VAC may be less affected by viral evolution than spike-based vaccines.

**Disclosures:**

**Anna Kushnir, PHD**, **Codagenix Inc** (Employee) **Steffen Mueller, PhD**, **Codagenix Inc** (Board Member, Employee, Shareholder) **Sybil Tasker, MD, MPH, FIDSA**, **Codagenix Inc** (Employee, Shareholder) **J. Robert Coleman, PhD**, **Codagenix Inc.** (Board Member, Employee, Shareholder)

